# Editorial: Modelling esophageal adenocarcinoma

**DOI:** 10.3389/fmolb.2024.1550595

**Published:** 2025-01-07

**Authors:** Hisham F. Bahmad, Farah Ballout

**Affiliations:** ^1^ Department of Pathology and Laboratory Medicine, University of Miami Miller School of Medicine, Miami, FL, United States; ^2^ Department of Surgery, Miller School of Medicine, University of Miami, Miami, FL, United States

**Keywords:** esophageal cancer, preclinical model, patient-derived organoid, liquid biopsy, machine learning

Esophageal cancer remains a significant global health concern with a persistently poor prognosis and high mortality rate (Sung et al., 2021). In 2024, it is estimated that approximately 22,370 new cases of esophageal cancer were diagnosed, and 16,130 deaths were attributed to the disease in the United States (Siegel et al., 2024). This Research Topic showcases a Research Topic of studies employing innovative strategies to address critical challenges in esophageal cancer research, including (1) the development of advanced research model systems, (2) discovery of novel biomarkers, and (3) application of machine learning to enhance diagnostic accuracy.

First, accurately recapitulating the complex biology of esophageal cancer in preclinical models is essential for advancing our understanding of disease pathogenesis and developing effective therapies. Two articles within this Research Topic highlight advancements in modeling esophageal adenocarcinoma and its precursor lesion, Barrett’s esophagus. Traditional research models, such as cell lines and genetically engineered animal models, often fall short of capturing the inherent heterogeneity of esophageal adenocarcinoma [Article 1 (*Esophageal adenocarcinoma models: a closer look*) and Article 3 (*Modelling esophageal adenocarcinoma and Barrett’s esophagus with patient-derived organoids*)] (Bhat et al.; Milne et al.). Patient-derived organoids offer a promising alternative, providing a more representative model for drug screening and mechanistic studies. The successful culture of Barrett’s esophagus patient-derived organoids provides a unique opportunity to investigate the transition of this precursor lesion to esophageal adenocarcinoma, a process that remains poorly understood in the disease’s pathogenesis.

Second, the identification of reliable biomarkers is critical for early detection and prognostication in esophageal cancer. The integration of network analysis with differential expression profiling revealed key genes in esophageal squamous cell carcinoma, such as *CDK1*, *MAD2L1*, *PLK1*, and *TOP2A*, which are implicated in cell survival and disease progression [Article 2 (*Integrating network analysis with differential expression to uncover therapeutic and prognostic biomarkers in esophageal squamous cell carcinoma*)] (Khurshid et al.). Moreover, *MMP9* emerged as a potential prognostic marker, with elevated expression correlating with poor survival outcomes. Similarly, a pan-cancer analysis identified *KCNN4* as a prognostic biomarker and a regulator of the tumor microenvironment, with potential implications for immunotherapy responsiveness [Article 7 (*KCNN4 is a Potential Biomarker for Predicting Cancer Prognosis and an Essential Molecule that Remodels Various Components in the Tumor Microenvironment: A Pan-Cancer Study*)] (Chen et al.). These findings collectively open the door for the development of targeted therapies and personalized treatments for patients.

Furthermore, liquid biopsies offer a minimally invasive approach for cancer detection and monitoring. The identification of a panel of blood-derived microRNAs (miRNAs) with stable expression patterns across multiple cancer types holds promise as a universal diagnostic tool [Article 6 (*A panel of blood-derived miRNAs with a stable expression pattern as a potential pan-cancer detection signature*)] (Sabbaghian et al.). By addressing the variability introduced by sampling times, this study establishes a framework for utilizing miRNAs as pan-cancer biomarkers, potentially enabling earlier and more accurate disease detection.

Third, the convergence of artificial intelligence and advanced technologies is rapidly transforming the landscape of medicine and research, offering unprecedented opportunities for precision diagnostics and personalized therapies. Machine learning, a powerful subset of AI, is playing a pivotal role in this revolution, particularly in the complex field of cancer diagnostics. This is clearly demonstrated by two studies within this Research Topic, which showcase the potential of machine learning in esophageal cancer. One study developed and validated automated machine learning models for predicting lymph node metastasis (LNM) in Siewert type II T1 adenocarcinoma of the esophagogastric junction [Article 4 (*The development and validation of automated machine learning models for predicting lymph node metastasis in Siewert type II T1 adenocarcinoma of the esophagogastric junction*)] (Lu et al.). Among the five models evaluated, the deep learning model demonstrated superior predictive performance, suggesting its utility in guiding clinical decision-making. Another study leveraged deep learning techniques to classify esophageal cancer subtypes using high-resolution histopathology images [Article 5 (*Deep learning-based identification of esophageal cancer subtypes through analysis of high-resolution histopathology images*)] (Aalam et al.).

The contributions to this Research Topic collectively underscore the importance of integrating advanced modeling systems, computational tools, and biomarker discovery in esophageal cancer research. The development of patient-derived organoids and deep learning models exemplifies how technological innovations are indeed bridging the existing gaps in our understanding of the different disease mechanisms and improving the diagnostic precision. Such findings should be validated in larger patient cohorts and translated into clinical applications. This Research Topic represents a step forward, providing a foundation for ongoing efforts to combat esophageal cancer. We thank all authors, reviewers, and editors who contributed to this compilation of cutting-edge research. Together, these studies illuminate new pathways for understanding and managing esophageal cancer, fostering hope for improved patient care and survival outcomes.
